# The Exosome-like Vesicles of *Giardia* Assemblages A, B, and E Are Involved in the Delivering of Distinct Small RNA from Parasite to Parasite

**DOI:** 10.3390/ijms24119559

**Published:** 2023-05-31

**Authors:** Lautaro Natali, Gabriel Luna Pizarro, Sofía Moyano, Benjamin de la Cruz-Thea, Juliana Musso, Andrea S. Rópolo, Norbert Eichner, Gunter Meister, Melina M. Musri, Constanza Feliziani, María C. Touz

**Affiliations:** 1 Instituto de Investigación Médica Mercedes y Martín Ferreyra, Consejo Nacional de Investigaciones Científicas y Técnicas (INIMEC-CONICET), Universidad Nacional de Córdoba, Córdoba 5016, Argentina; lnatali@immf.uncor.edu (L.N.); glpizarro@immf.uncor.edu (G.L.P.);; 2 Regensburg Center for Biochemistry (RCB), Laboratory for RNA Biology, University of Regensburg, 93053 Regensburg, Germany

**Keywords:** *Giardia lamblia*, parasite to parasite communication, exosomal-like vesicles (ElVs), small RNAs (sRNA), msRNA, tsRNA, rsRNA, trophozoites, host-specificity, pathogenesis

## Abstract

The genetically related assemblages of the intestinal protozoa parasite *Giardia lamblia* are morphologically indistinguishable and are often derived from specific hosts. The *Giardia* assemblages are separated by large genetic distances, which might account for their relevant biological and pathogenic differences. In this work, we analyzed the RNAs cargo released into exosomal-like vesicles (ElVs) by the assemblages A and B, which differentially infect humans, and the assemblage E, which infects hoofed animals. The RNA sequencing analysis revealed that the ElVs of each assemblage contained distinct small RNA (sRNA) biotypes, suggesting a preference for specific packaging in each assemblage. These sRNAs were classified into three categories, ribosomal-small RNAs (rsRNAs), messenger-small RNAs (msRNAs), and transfer-small RNAs (tsRNAs), which may play a regulatory role in parasite communication and contribute to host-specificity and pathogenesis. Uptake experiments showed, for the first time, that ElVs were successfully internalized by the parasite trophozoites. Furthermore, we observed that the sRNAs contained inside these ElVs were first located below the plasma membrane but then distributed along the cytoplasm. Overall, the study provides new insights into the molecular mechanisms underlying the host-specificity and pathogenesis of *G. lamblia* and highlights the potential role of sRNAs in parasite communication and regulation.

## 1. Introduction

The genus *Giardia* has been classified into six species or assemblies depending on the host, with *Giardia lamblia* (syn. *G. intestinalis*, *G. duodenalis*) being the only specie found infecting humans (assemblages A and B). *G. lamblia* also infects a broad range of other mammals including wild and domestic animals and causes giardiasis, a disease also related to zoonotic transmission [1]. The host specificity is important when distinguishing different species because the morphology and ultrastructure of the parasites are very similar. For instance, the assemblages C and D infect dogs; E infects cattle, sheep, goats, pigs, and water buffaloes; F infects cats; and G is restricted to rodents [2]. From the data obtained so far, it is strongly suggested that the assemblages A and B are different species [3]. Using comparative proteomics, differences were found in several proteins between *Giardia* isolates from assemblages A and B [4]. Additionally, differences in metabolism, nucleotide content, in vitro and in vivo growth rates, drug sensitivity, and clinical features, among other characteristics, have been reported between both assemblages [5,6,7,8,9]. Some groups have shown that several differences in excretory-secretory products (ESPs), including extracellular vesicles (EVs), occurred between assemblage A and B trophozoites, which altered the gene expression of intestinal epithelial cells (IECs) when interacted in vitro [10,11]. For instance, it was shown that the interaction of the two assemblages with IECs led to distinct antioxidative reactions [11]. Interestingly, the dominant proteins that trigger an immune response during an infection, known as Variable Surface Proteins (VSPs), along with a significant amount of High Cysteine Membrane Proteins (HCMPs), were found in the ESPs of the assemblage A isolate. Conversely, fewer VSPs and HCMPs were detected in the assemblage B isolates ESPs [11]. More recently, EVs were isolated from both assemblages and subjected to nanoparticle tracking and proteomic analysis, revealing two types of EVs: small vesicles (exosome-like particles, <100 nm) and large vesicles (microvesicles-like particles, 100–400 nm) [12]. Typical rounded or cup-shaped vesicles with a membrane bilayer of around 100~200 nm were also reported for Assemblage A [13,14]. Regarding EVs, they have emerged as a ubiquitous mechanism for transferring information between cells and organisms across the three kingdoms of life, serving as vehicles for the transfer of cytosolic and membrane proteins, lipids, and RNAs [15,16,17]. In parasites, EVs are regularly released during different stages of development and, in addition to their functions in normal physiology, they also transport virulence factors to hosts and can spread antigens and infectious agents [18,19,20]. The RNA cargo composition of EVs involves various biotypes, such as small non-coding RNAs, fragmented and intact messenger RNAs (mRNAs), ribosomal RNAs (rRNAs), and long non-coding RNAs (lncRNAs). [21]. Little is known about the biogenesis and regulation of small RNAs (sRNAs) with a size less than 40 nt in *Giardia*, although several sRNAs have been identified [22,23]. Moreover, the sRNAs cargo composition of giardial EVs is unknown, but it is possible that it differs from the content of the sRNAs found in growing and encysting trophozoites as it was found in other cells [24,25,26]. To disclose whether sRNAs packaging into EVs is a selective process, we performed a careful analysis of the sequencing data and experimental verification of the sRNA found in exosomal-like vesicles (ElVs) derived from assemblages A, B, and E. We demonstrated that all EIVs exhibited the size and shape of exosomes and were enriched with small RNAs, including ribosomal-small RNAs (rsRNAs), messenger-small RNAs (msRNAs), and transfer-small RNAs (tsRNAs). We observed active internalization of the specifically labeled sRNAs contained in the EIVs, followed by cytoplasmic localization, after exposing them to trophozoites from all three assemblages. As sRNAs delivered in EVs are involved in diverse biological functions such as gene regulation and post-transcriptional control [27], we propose that this intercellular communication may be a possible mechanism used by the parasite to ensure its survival inside the host. In this work, we refer to *Giardia* assemblages as groups that are genetically distinct within the specie, while we will refer to isolates (e.g., WB/1267, GS/H7, and P15) as those obtained from patients, which belong to different assemblages, and that we use to study sRNA differences and cellular behavior.

## 2. Results

### 2.1. The Assemblages A, B, and E of Giardia Release Exosome-like Vesicles Containing Small RNAs

**Figure 1:**
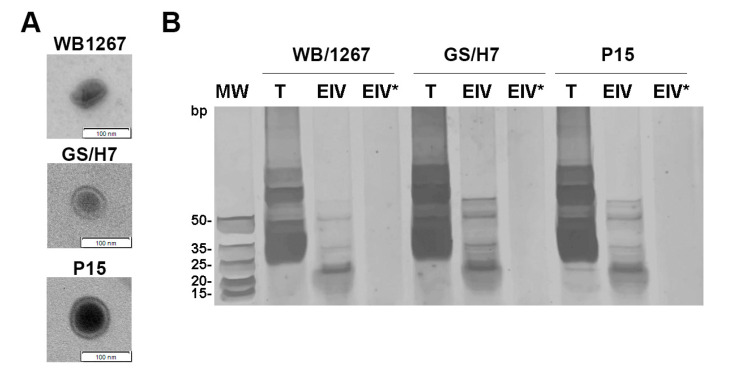
The *Giardia* isolates WB/1267, GS/H7, and P15 produce exosomal-like vesicles enriched in small RNAs. (**A**) Negative staining electron microscopy shows cup-shaped vesicles. Bar: 100 nm. (**B**) Silver staining of total RNA extracted from trophozoites (T), ElVs (ElV), and ElVs treated with Triton-X100 and RNAse A (ElV*) for each isolate. MW is shown on the **left**.

Using a combination of biochemical and cell biology analyses, we recently showed that *G. lamblia* releases ElVs of similar size, shape, and protein and lipid composition to that described for exosomes from other eukaryotic cells [14]. To analyze the RNA content of the ElVs released for each assemblage, we first examined whether the enriched fractions of ElVs showed vesicles of similar shape and size for each assemblage. As shown in Figure 1A, the ElVs purified from the isolates WB/1267 (Assemblage A), GS/H7 (Assemblage B), and P15 (Assemblage E) showed a range size of 70 to 100 nm with a cup-shaped morphology being consistent with the ElVs we reported previously for WB/1267 [14]. Although the analysis of the protein components of the secretome of assemblages A and B has been published [10,28,29], the RNAs cargo has not yet been described. To obtain an overview of the RNA repertoire, duplicate samples of total RNA from trophozoites and enriched ElVs were separated on a denaturing polyacrylamide gel. The results showed a higher yield of RNAs of different sizes from trophozoite lysates (T) compared to ElVs. However, the three ElV samples (ElV) displayed a specific enrichment of sRNAs (Figure 1B).

To confirm whether the small RNAs were truly packaged into ElVs, isolated ElVs were subjected to treatment with Triton X-100 followed by RNAse A (ElV*). The results showed that this population of sRNAs was indeed packaged inside membrane-bound vesicles and protected from degradation by the ElVs (Figure 1B).

### 2.2. RNA-Seq of Giardia ElVs Revealed the Presence of Different sRNA Biotypes

**Figure 2:**
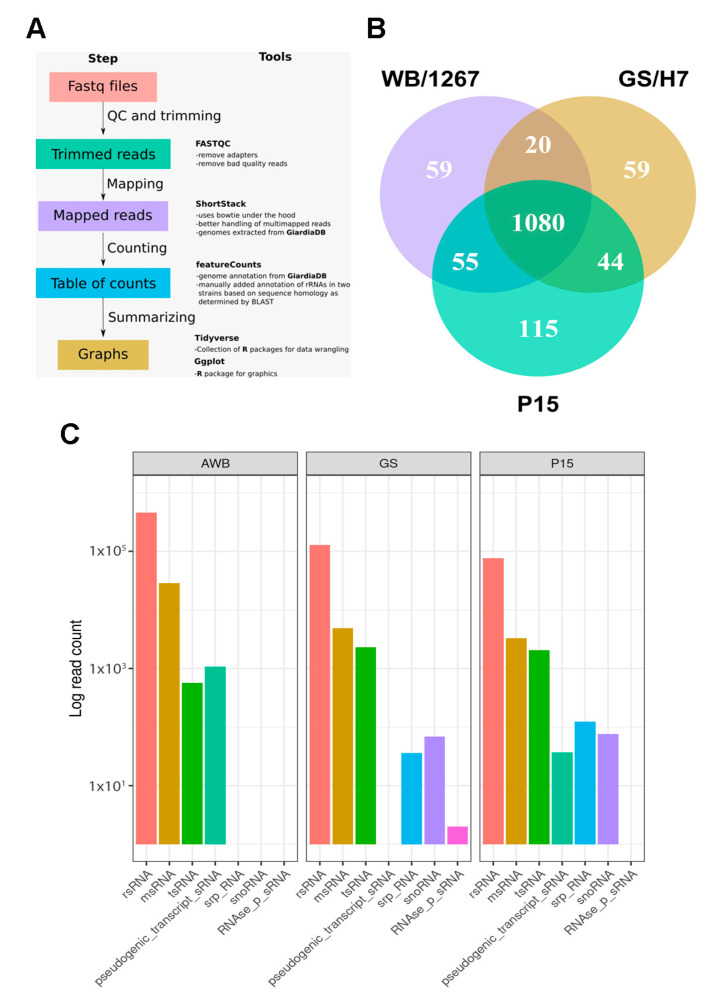
Functional categorization of sequencing reads from small RNA-seq libraries of ElVs of the isolates WB/1267, GS/H7, and P15 of *Giardia*. (**A**) A scheme of the bioinformatic data performed after sequencing for all isolates is presented. (**B**) The Venn diagram illustrates all sRNAs identified in ElVs from WB/1267, GS/H7, and P15 of *Giardia* isolates. (**C**) The biotypes of sRNA are based on *G. lamblia* genome annotations.

To characterize the RNA cargo of ElVs, we performed deep sequencing of small RNAs from isolated vesicles, as detailed in the Materials and Methods section. To assess reproducibility, we prepared three small RNA libraries from two independent preparations of *Giardia* ElVs. Standard bioinformatics tools were used to map and assign the reads to RNA biotypes and to identify putative small RNAs (Figure 2A and Materials and Methods section). We observed unique and shared sequences between the three assemblages (Figure 2B), suggesting that small RNAs can be selectively packaged into ElVs. We also identified de novo RNA expression clusters, which represented the most abundant transcripts in each isolate, using the Shortstack pipeline [30].

These sequences were then used to perform a specific alignment within the genome and transcriptome of each isolate using Blastn [31], which allowed us to identify the origin of most clusters and the genes to which they correspond. Additionally, we used a reference-based approach by entering the Binary Alignment (BAM) files generated by Shortstack into the featureCounts program of the Rsubread package in the R environment. The program identified the already annotated genes to which all the reads mapped by ShortStack corresponded, irrespective of their participation in expression clusters. Next, a summary table grouping the sRNAs by biotypes was generated in the R environment, enabling comparisons between isolates (Table S1). Analysis using miRDeep (an integrated application tool for micro RNA—miRNA—identification from RNA sequencing data) [32], did not allow us to identify miRNA-like sequences from either *Giardia* or other species (see Discussion). Although we were unable to detect representative miRNAs, our analysis showed several sRNA sequence reads of rsRNAs, tsRNAs, and msRNAs for all three isolates, with differences in the number of representative sequences for each (Figure 2C). Our results showed that the rsRNAs constituted the majority of reads among the three isolates analyzed, with the WB/1267 isolate having the highest raw number of reads. Although 76.4% of the sRNAs were shared between all isolates (Figure 2B), there were also sRNAs exclusive to each isolate (4.1–7.7%) and shared between pairs (1.3–3.7%). Although statistical comparisons were not possible due to methodological limitations, some notable differences in the principal sRNAs detected in each sample were observed. In particular, the WB isolate exhibited high expression levels of VSPs, which was among the top five most expressed sRNAs, whereas the other isolates showed only minimal expression of VSP.

### 2.3. The ElVs of the Assemblages A, B, and E of Giardia Contain Unique and Shared Small RNAs

RT-PCR was applied to confirm the presence of shared as well as unique RNA fragments of the ElVs from the three isolates. Using reverse complementary probes to their identical 5′ end sequence, we analyzed rsRNA-28S, present in the ElVs of the three isolated, as well as tsRNA-Gln, tsRNA-Pro, and tsRNA-Ser for isolates WB1267, GS/H7, and P15, respectively. We used RT-PCR to confirm the presence of shared as well as unique RNA fragments of the ElVs from the three isolates. To analyze rsRNA-28S, present in the ElVs of all three isolates, we used reverse complementary probes to their identical 5′ end sequence (Table 1).

qPCR identified small RNAs from *Giardia* trophozoites and ElVs. The results showed common rsRNAs as well as unique tsRNA and msRNAs in the isolates. The most abundant fragments from each isolate were selected and are presented in Table 2. Despite improvements to the qPCR assay, we detected tsRNA-Pro amplification in negative and positive controls of trophozoites and ElVs from the GS/H7 isolate. This non-specific amplification might be due to the primers having a high degree of homology to other RNA sequences in the sample or due to off-target effects.

### 2.4. The Extracellular Vesicle RNA Cargo Is Internalized by the Trophozoites

**Figure 3:**
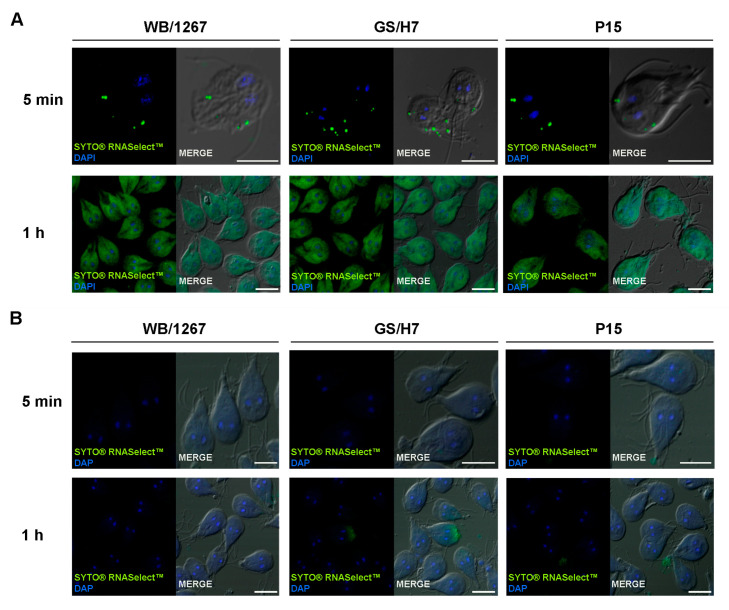
The sRNA contained in the ElVs can be observed inside the *Giardia* trophozoites. (**A**) Confocal microscopy images show trophozoites from the isolates WB/1267, GS/H7, and P15 incubated for 5 min or 1 h with their corresponding SYTO^®^ RNASelect™-labelled ElVs (green). (**B**) Trophozoites incubated with free-ElVs labelled samples do not show RNA internalization. Confocal microscopy images show trophozoites from the isolates WB/1267, GS/H7, and P15 incubated for 5 min or 1 h with free-ElVs sample with SYTO^®^ RNASelect™ (green). 4 6-Diamidino-2-phenylindole (DAPI) stains the nuclei (blue). Overlaying fluorescence and differential interference contrast (DIC) images are shown in the merge panels. Bars, 5.0 μm. Images are representative of three independent experiments with *n* = 50 trophozoites.

Previous studies have demonstrated that incubating differentiated Caco-2 cells with either large or small EVs from *Giardia* trophozoites or with the whole secretome can affect the gene expression of the host cells [10,28,29]. However, communication between trophozoites has not been reported until now. Although the molecular mechanism for ElV uptake by recipient cells is unknown, the most common mechanism of internalization involves endocytosis, whereby extracellular vesicles are taken up into endosomes [33]. In the endosome, RNA content might be released into the luminal space or into the cytoplasm. To track the RNA delivery into the trophozoites, we labeled the ElV RNA using the SYTO^®^ RNASelect™ Green Fluorescent Cell Stain reagent. This compound is a cell-permeant nucleic acid stain that is highly selective for RNA since it is nonfluorescent in the absence of nucleic acids, exhibits bright green fluorescence when bound to RNA, and only a weak fluorescent signal when bound to DNA. This stain very efficiently labels the exosomal RNA cargo [34]. After labeling, ElVs were incubated with their belonging trophozoites for 5 min or 1 h. At 5 min, green spots similar to the localization of the endosomal-lysosomal peripheral vacuoles (PVs) situated below the plasma membrane were observed, indicating effective EIV internalization (Figure 3A). Later, the green-labeled sRNAs were observed on the cytoplasm of the trophozoites (Figure 3A). The same amount of PBS (100 µL) without ElVs were treated with SYTO^®^ RNASelect™ and used as control for specific ElVs uptake (Figure 3B). The results showed the lack of green labelling on the trophozoites treated with the control samples, strongly indicating that the RNA was only efficiently delivered to the trophozoites via the ElVs.

## 3. Discussion

The unique biology and roles of extracellular vesicles (EVs) in cell-to-cell communication have attracted strong interest due to their increasing utility in studying cell behavior and potential clinical applications. For extracellular parasites, the impact of EVs has been analyzed, showing an important function in modulating the host immune system [35,36,37,38,39,40,41,42]. However, how extracellular parasites communicate with each other to ensure their survival, either by triggering cell differentiation, regulating protein expression, and/or changing their metabolism according to the environment’s requirements, is less explored. In this sense, an important role is played by the RNA cargo, which can elicit its functional effect when it reaches the cytoplasm of another cell. For example, small interfering RNAs (siRNAs) can inhibit the translation of specific transcripts, whereas messenger msRNAs can be translated into functional proteins [43].

The present work aims to demonstrate that the ElVs produced by *Giardia*’s assemblages A, B, and C have the size and shape of exosomes and are enriched with rsRNA, msRNA, and tsRNA. Furthermore, we have made progress in observing the delivery of cargo by extracellular vesicles to recipient trophozoites in these three assemblages, although the mechanism behind this process remains unclear. Additionally, we were able to identify and compare similar and specific sRNAs that are trafficked in each delivered EV.

The biological properties of isolates belonging to different assemblages have shown differences in terms of relative infectivity, host-specificity, and zoonotic potential [44,45]. Additionally, there are assemblage-specific differences in the efficiency of encystation in vitro [46,47]. As *Giardia* might be considered a complex species, we intended to analyze whether different assemblages possess a mechanism of extracellular vesicle production and release that might explain these differences. We observed that assemblages A, B, and E released cup-shaped EVs with an average size of 80 nm, consistent with the description of the smallest extracellular vesicles produced by most cells [17]. This agrees with our previous report and with other investigations showing the host-cell acquisition of EVs from the culture supernatants of assemblages A and B [10,28]. In this work, we also showed that the ElVs are enriched with sRNAs, which might be protected from degradation outside the trophozoite when they are packaged into these vesicles. It is possible that long RNAs could be incorporated in a condensed configuration [48], and their absence in the polyacrylamide gel of this work might reflect their relatively low abundance in the giardial EVs. Deep sequencing revealed that the sRNAs from the three assemblages are enriched in msRNA, rsRNA, and tsRNA. Interestingly, the majority of the rsRNA fragments were derived from the large 28S ribosomal subunit sequences, whereas a lower quantity corresponded to the 18S small subunit fragments. In this sense, the overall level of rsRNA fragments in EVs is too high to be attributed to contaminating factors. However, it is possible that other sRNAs, such as snoRNA or sRNAse, were underrepresented because the rsRNA was not removed in our assays and may have masked their presence. rsRNA fragmentation products are now known to be prevalent in all EVs [49,50] and play essential roles in cellular communication via extracellular vesicles [49,50,51,52]. A similar case occurs for tsRNA [53]. To date, a wide variety of tRNA-derived fragments, such as tRNA fragments and tsRNAs, have been identified, some with the direct ability to inhibit protein synthesis and function as miRNAs [54]. Additionally, the miRNA and tsRNA pathways may be linked to the mechanism involving Dicer and Argonaute [55,56]. In this study, we found a predominance of vesicular tsRNA-Gly, tsRNA-Gln, and tsRNA-Arg for assemblages A, B, and E, respectively. Previous research has shown that trophozoites of assemblage A produced tRNA-derived sRNAs and proposed that they might be associated with the downregulation of gene expression [57].

The relative abundance of tsRNAs in the EVs of the three assemblages is consistent with previous findings in other protozoan parasites [54,58,59,60,61]. However, it has yet to be determined whether these tsRNA fragments contribute to any biological functions in these parasites. Additionally, an interesting finding was that msRNAs in EVs could serve as a source of novel proteins in recipient cells [62,63].

In this work, the presence of a high amount of msRNA belonging to hypothetical proteins is not surprising, since according to the *Giardia* database (GDB), 70% of the *Giardia* genome comprises non-conserved protein genes. However, this study observed the ElVs-mediated transfer of msRNAs of the actin cytoskeleton regulators, metabolic regulators, and extracellular matrix remodeling, which agrees with reports in other eukaryotic cells [64]. Importantly, the ElVs transported by assemblage A carried a larger amount of msRNA for VSPs, suggesting that these ElVs might be implicated in the control of antigenic variation in the recipient cells of this assemblage. It has been shown that the secretome of the coculture of IECs with trophozoites of assemblage A contained many VSPs, the primary proteins that stimulate the immune system during infection, whereas the secretome of assemblage B displayed fewer VSPs [11]. Although the differential secretion of VSPs and msRNA of VSPs can be attributed to the utilization of a fragmented genome in assemblages B and E, our finding alerts for a possible assemblage-specific VSPs switching that helps these parasites better evade the host immune system.

It was not possible to identify miRNA in any ElVs using the prediction of their canonical structure by miRDeep, which was not surprising given that *Giardia* lacks proteins that participate in miRNA processing, such as DROSHA and XPO5 [65,66]. This points to the possibility that this parasite possesses a non-canonical miRNA generation pathway, as has been suggested [65]. Furthermore, the comparison of the sequences with the few known miRNAs present in the *Giardia* trophozoites from assemblage A was not successful, possibly due to the lack of data available in this nascent field [65,66].

sRNA cargo delivery by EVs can be facilitated by using and tracking labeled RNAs in recipient cells. Our results showed that trophozoites internalized fluorescent-green sRNA from ElVs belonging to the same isolate. Immediately upon entering the trophozoites, the RNA seemed to be localized below the plasma membrane in the endo-lysosomal PVs, similar to reports of endosomal-sRNA localization in other cells [67]. Later, the sRNAs were localized in the trophozoites’ cytoplasm, suggesting an active mechanism for PVs escape. In the cytoplasm, the sRNA cargo might reach its functional effect. Further research is underway to determine whether the differential packaging of sRNA and proteins in ElVs is used by each assemblage to establish host-specific infection and parasite pathogenicity.

## 4. Materials and Methods

### 4.1. Cell Culture and EV Isolation

Axenic cultures of *Giardia* trophozoites of isolates WB clone 1267 (ATCC 50582, Assemblage A), GS/M (ATCC 50581, Assemblage BIV) were purchased at (www.atcc.org, accessed on 1 March 2008). The P15 isolate (Assemblage E) was originally isolated in the Czech Republic from the upper jejunum of a necropsied piglet that was naturally infected with *Giardia* [68]. Trophozoites were routinely grown in 16 mL screw-cap tubes (NuncTM, ThermoFisher Scientific, Waltham, MA, USA) in TYI-S33 medium, supplemented with 10% adult bovine serum (Euroclone, Pero, Italy) and bovine bile (Sigma-Aldrich S.R.L., Milan, Italy) [69] at 37 °C. Log-phase cultures were harvested after cooling the culture vials on ice for 15 min and centrifugation at 700× *g* for 10 min. Enriched exosome-like vesicles (ElVs) were obtained using differential ultracentrifugation from the supernatant of trophozoites, as we described [14], modified from Thery et al., 2006 [70]. Briefly, 14 × 107 trophozoites recovered from the monolayer were washed twice with warm PBS 1X (37 °C). To avoid exosomal contamination from other sources, the trophozoites were incubated in TYI-S-33 medium without serum and without bovine bile (TYI-S-33/-sbb) for 4 h at 37 °C prior to isolation. Then, the parasites were removed by centrifugation at 1455× *g* for 15 min and the supernatant recovered. After centrifugation, the supernatant was filtered through a 0.11-µm filter (Millipore) to discard high-size vesicles. To obtain the exosomal fraction, the filtered elution was subsequently pelleted at 100,000× *g* for 200 min using a 60Ti rotor (Beckman-coulter L-70 Ultracentrifuge). The pellet was then washed with PBS and pelleted again at 100,000× *g* in the same ultracentrifuge.

### 4.2. Exosome-like Vesicles (ElVs) Size and Electron-Microscopy Images

For negative electron microscopy staining, ElVs were diluted in PBS, placed on copper grids, and incubated 15 min at room temperature. Liquid excess was removed by blotting. The grid was placed on 2% uranyl acetate (*w*/*v*) (Merck, Darmstadt, Germany) for 30 s and observed in a JEOL 1230 transmission electron microscope, as performed before [14].

### 4.3. ElVs RNA Profiling

RNA profiling was performed on lysate of trophozoites, enriched ElVs, and ElVs treated with Triton X-100 and RNase A using the Total Exosome RNA Isolation Kit (Invitrogen) and following the manufacturer’s instructions available at as-sets, https://assets.thermofisher.com/TFS-Assets/LSG/manuals/total_exosome_kit_man.pdf, accessed on 31 May 2022. This kit utilizes AcidPhenol:Chloroform extraction to provide a robust front-end RNA purification step, followed by a final RNA purification over a glass-fiber filter [71]. RNA concentrations were determined using the NanoDrop 2000 spectrophotometer (Thermo Fisher Scientific, Waltham, MA, USA). To compare RNA profiles, a 15% denaturing polyacrylamide gel using 8 M urea and 1× TBE was utilized. The samples were loaded in denaturing buffer and run at 100 V in 1× TBE buffer for 3 h. The gel was visualized using a silver staining kit (Fermentas) following the manufacturer’s protocol. To assess the internalization of the RNA cargo within vesicles, isolated ElVs were briefly vortexed after treatment with 0.1% Triton X-100 and subsequently incubated with RNase A (10 mg/mL) for 15 min at 37 °C. After treatment, RNA was purified as indicated above. The GeneRuler™ Ultra Low Range DNA Ladder (Invitrogen) was used as molecular weight standard.

### 4.4. Small RNA Libraries and RNA-Seq

The sRNA fraction (200 ng) obtained from *Giardia* trophozoites ElVs, without any enzymatic treatment or rRNA depletion step, was used for library preparation following the manufacturer’s instructions (NEBNext_ Small RNA Library Prep Set for Illumina from New England BioLabs, MA, USA). For deep sequencing, the library generation protocol was performed according to the AQ Seq protocol from the lab of Narry Kim [72,73], with minor modifications. In brief, total RNA was ligated with randomized 3′ adapter using T4 RNA ligase 2 truncated KQ (NEB) in 1X T4 RNA ligase reaction buffer (NEB) supplemented with 20% PEG8000 (NEB). The ligated miRNA-adapter fragments were gel-purified and eluted in 300 mM NaCl, 2 mM EDTA (pH 8). After centrifugation, the EtOH-precipitated fragments were resuspended in water and ligated to 0.25 µM randomized 5′ RNA adapter using T4 RNA ligase 1 (NEB) in the same buffer conditions as described above. Reverse transcription was performed with Superscript III First-Strand synthesis supermix (Thermo Fisher Scientific) and 5 µM RT Primer (“RTP”; Illumina) according to the manufacturer’s recommendations. After cDNA amplification, the PCR amplicons representing miRNA-sized inserts were gel-purified again, precipitated, and finally resuspended in water. The quality of the libraries was assessed by qPCR and Tapestation measurements, and the pooled libraries were sequenced on a Nextseq 550 sequencer (Illumina) as an 80-cycle single-end run.

### 4.5. Bioinformatic Analysis

The RNA reads obtained from sequencing were trimmed to remove the adapters and then mapped to the genomes of each of the *Giardia* isolates in FASTA format (GiardiaDB: https://giardiadb.org/giardiadb/app/, accessed on 10 June 2022) using ShortStack [30] to identify small groups or clusters of expression. This tool uses the Bowtie aligner [74] for mapping and then a proprietary algorithm to work with reads that map to more than one locus in each genome, improving the detection of small RNA (sRNAs) originating from repeated sequences of the genome, such as ribosomal-small RNAs (rsRNAs) and transfer RNAs (tsRNAs). The de novo clusters of RNA expression were identified using ShortStack [30]. The most abundant sequence was then used to carry out searches with Blastn [31] in the genomes and transcriptomes of each isolate. This allowed the localization of the origin of these clusters and the genes to which they correspond if they are listed. In addition to the de novo identification of clusters, a reference-based approach was used. To do this, the alignments in BAM (Binary Alignment) format generated by Shortstack were introduced to the featureCounts [75] program of the Rsubread package in the “R” software environment. The program identified the annotated genes to which all the reads mapped by ShortStack corresponded, regardless of their participation or not in expression clusters, generating a summary table that allowed grouping them later by biotypes to make comparisons between assemblages, also in the “R” environment.

### 4.6. PCR Detection and Quantification of Small RNAs

To confirm the presence of sRNAs in ElVs, RNA was isolated from both trophozoites and freshly isolated ElVs using the Total Exosome RNA and Protein Isolation kit, following the manufacturer’s protocol (Thermo Fisher Scientific Inc.). RT-qPCR was performed to verify the selected RNA targets identified from the RNA-seq analysis (Table 1). Briefly, two micrograms of total RNA from each sample were reverse transcribed with Reverse Transcriptase M-MLV (Promega) and the specific Rloop reverse primer (Table 2). The resulting cDNA was analyzed by real-time PCR with SYBR Green Master Mix (Thermo Fisher Scientific Inc.), using 100 ng of the input total RNA equivalent as single-stranded cDNA and 800 nm of amplification primer in a reaction volume of 20 μL. Primers used in this study are listed in Table 3. The runs were conducted on a standard 7500 system (Applied Biosystems). The qPCR was performed using the Common Reverse Primer and a forward primer specific to the target small RNA (see below). The RT-qPCR conditions were 50 °C for 2 min; 95 °C for 510 min; and 40 cycles at 95 °C for 15 s; and 60 °C for 10 s and 72 °C for 1 s min after 95 °C for 15 s; 60 °C for 1 min; and 95 °C for 14 s. Recorded cycle threshold (Ct) values were descriptively compared. These assays were performed in duplicates. All DNA oligonucleotides were purchased from Macrogen (Macrogen, Seoul, Republic of Korea).

### 4.7. Labelling the RNA from EVs

ElVs were labelled with RNA-specific dye SYTO^®^ RNASelect™ (Thermo Fisher Scientific Inc.) according to the manufacturer’s recommendations, except that removal of excess dye was performed by ultracentrifugation. Briefly, enriched ElVs from the trophozoites were suspended in 100 µL of PBS per labelling reaction. Then, 1 µL of the 1 mM dye stock solution was added to the samples and mixed before incubating at 37 °C for 20 min, protected from light. To remove the excess of unincorporated dye, two rounds of ultracentrifugation at 100,000× *g* for 200 min using a 60Ti rotor (Beckman-coulter L-70 Ultracentrifuge) were performed. The pellet containing the freshly labelled ElVs was recovered and used in uptake assays. As control for unspecific ElVs RNA staining, the same procedure was performed on samples containing SYTO^®^ RNASelect™ but lacking the ElVs. Briefly, 1 µL of the 1 mM dye stock solution was added to 100 µL of PBS per labelling reaction and mixed before incubating at 37 °C for 20 min, protected from light. Later, two rounds of ultracentrifugation at 100,000× *g* for 200 min using a 60Ti rotor (Beckman-coulter L-70 Ultracentrifuge) were performed. After washing, the fraction from the bottom of the tube was recovered and used for uptake experiments.

### 4.8. Cell Uptake Assays

100,000 trophozoites of each assemblage were incubated with the corresponding fluorescently labeled ELVs at 37 °C for 5 min (to trace early incorporation of RNA) or 1 h (to observe the final destination of RNA inside the cell). The same volume of PBS without ELVs, treated with SYTO RNA, was used as a control. After washing with PBS, the cells were fixed with 4% paraformaldehyde at room temperature for 20 min and then permeabilized with 0.1% Triton X-100 (Sigma) at room temperature for 5 min, following the protocol described by the company (https://assets.thermofisher.com/TFS-Assets/LSG/Application-Notes/Exosome%20Tracing_App%20Note.pdf, accessed on 12 November 2021). After two washes, the trophozoites were incubated with DAPI and mounted using FluoSafe^®^ (Sigma). Fluorescence staining was visualized with a motorized FV1200 Olympus confocal microscope (Olympus UK Ltd., Southend-on-Sea, UK) using 63× or 100× oil immersion objectives (numerical aperture 1.32). Differential interference contrast images were collected simultaneously with the fluorescence images using a transmitted light detector. The images were processed using Fiji software [76].

## Figures and Tables

**Table 1:** Selected common and unique sRNAs for primer design and qRT-PCR assay.

Isolate	sRNA	Sequence	*GiardiaDB*-ID
WB/1267	rsRNA-28S	CCCGCGGAACGGCGUCGG	GL50803_r0021
GS/H7	rsRNA-28S	CCCGCGGAACGGCGUCGG	GL50803_r0021 ^1^
P15	rsRNA-28S	CCCGCGGAACGGCGUCGG	GL50803_r0021 ^1^
WB/1267	tsRNA-Gln	UGGGGCGUGGUGCAGCGGGAGC	GL50803_t0051
GS/H7	tsRNA-Pro	CUUUGGGUGCGAGAGGCC	GL50581_4624
P15	tsRNA-Ser	UGGGGCGUGGUGCAGCGGGAGC	GLP15_4341
WB/1267	msRNA-VSP	CGACUGCCCGAUCGAGAACUGC	GL50803_3327
GS/H7	msRNA-HP	CUCUUGUAGACCGUCGCU	GL50581_1332
P15	msRNA-IF	AUGUGAGAGUAGGUCAUC	GLP15_4980

^1^ Gene ID was predicted from the annotated GL50803 Assemblage. VSP: Variant-specific protein; HP: Hypothetical protein; IF: Translation initiation factor.

**Table 2:** Recorded cycle threshold (Ct) values of the assemblage-specific qRT-PCR assays.

Isolate	sRNA	Sample	Ct Value ± SD
WB/1267	rsRNA-28S	Trophozoites’ RNA ElVs’ RNA	33.09 ± 1.89 35.72 ± 1.98
	tsRNA-Gln	Trophozoites’ RNA ElVs’ RNA	26.41 ± 1.70 28.99 ± 1.61
	msRNA-VSP	Trophozoites’ RNA ElVs’ RNA	29.25 ± 1.71 30.70 ± 1.78
GS/H7	rsRNA-28S	Trophozoites’ RNA ElVs’ RNA	33.50 ± 0.97 35.67 ± 1.31
	tsRNA-Pro	Trophozoites’ RNA ElVs’ RNA	- -
	msRNA-HP	Trophozoites’ RNA ElVs’ RNA	35.95 ± 0.53 19.10 ± 0.53
P15	rsRNA-28S	Trophozoites’ RNA ElVs’ RNA	33.50 ± 0.90 33.62 ± 1.05
	tsRNA-Ser	Trophozoites’ RNA ElVs’ RNA	22.46 ± 0.70 29.05 ± 0.37
	msRNA- IF	Trophozoites’ RNA ElVs’ RNA	36.80 ± 0.37 31.34 ± 2.08

SD: standard deviation. VSP: Variant-specific protein; HP: Hypothetical protein; IF: Translation initiation factor.

**Table 3:** qRT-PCR primers.

Name	sRNA	Sequence
R1loop	rsRNA-28S	GTCGTATCCAGTGCAGGGTCCGAGGTATTCGCACTGGATACGACGGCCGA
F1	rsRNA-28S	TAATAAGGCCCGCGGAACG
R2loop	tsRNAGly/tsRNASer	GTCGTATCCAGTGCAGGGTCCGAGGTATTCGCACTGGATACGACGCTCCC
F2	tsRNAGly/tsRNASer	ATAGTATGGGGCGTGGTGC
R3loop	tsRNA-Pro	GTCGTATCCAGTG GTCGTATCCAGTGCAGGGTCCGAGGTATTCGCACTGGATACGACGGCCTC
F3	tsRNA-Pro	AAGAATTCGCTTTGGGTGCG
R4loop	msRNA-VSP	GTCGTATCCAGTGCAGGGTCCGAGGTATTCGCACTGGATACGACGCAGTT
F4	msRNA-VSP	AACTTCCGACTGCCCGATC
R5loop	msRNA-HP	GTCGTATCCAGTGCAGGGTCCGAGGTATTCGCACTGGATACGACCAAGCG
F5	msRNA-HP	AACCACTGACTCTTGTAGAC
R6loop	msRNA- IF	GTCGTATCCAGTGCAGGGTCCGAGGTATTCGCACTGGATACGACTCGATG
F6	msRNA- IF	AAGCGACCAGATGTGAGAGTAG
Common Reverse Primer	-	GTCGTATCCAGTGCAGGGT

VSP: Variant-specific protein; HP: Hypothetical protein; IF: Translation initiation factor.

## Data Availability

The data presented in this study are available in this article and in the Supplementary Material here. More data are available on request from the corresponding author.

## Supplementary Materials

The supporting information can be downloaded at: https://www.mdpi.com/article/10.3390/ijms24119559/s1.
